# Spatial, Temporal, and Species Variation in Prevalence of Influenza A Viruses in Wild Migratory Birds

**DOI:** 10.1371/journal.ppat.0030061

**Published:** 2007-05-11

**Authors:** Vincent J Munster, Chantal Baas, Pascal Lexmond, Jonas Waldenström, Anders Wallensten, Thord Fransson, Guus F Rimmelzwaan, Walter E. P Beyer, Martin Schutten, Björn Olsen, Albert D. M. E Osterhaus, Ron A. M Fouchier

**Affiliations:** 1 Department of Virology, Erasmus Medical Center, Rotterdam, The Netherlands; 2 Section for Zoonotic Ecology and Epidemiology, Department of Biology and Environmental Science, University of Kalmar, Kalmar, Sweden; 3 Smedby Health Center, Kalmar County Council, Kalmar, Sweden; 4 Division of Medical Microbiology, Department of Molecular and Clinical Medicine, Faculty of Health Sciences, Linköping University, Linköping, Sweden; 5 Bird Ringing Center, Swedish Museum of Natural History, Stockholm, Sweden; 6 Department of Clinical Science, Uppsala University Hospital, Uppsala, Sweden; University of Wisconsin-Madison, United States of America

## Abstract

Although extensive data exist on avian influenza in wild birds in North America, limited information is available from elsewhere, including Europe. Here, molecular diagnostic tools were employed for high-throughput surveillance of migratory birds, as an alternative to classical labor-intensive methods of virus isolation in eggs. This study included 36,809 samples from 323 bird species belonging to 18 orders, of which only 25 species of three orders were positive for influenza A virus. Information on species, locations, and timing is provided for all samples tested. Seven previously unknown host species for avian influenza virus were identified: barnacle goose, bean goose, brent goose, pink-footed goose, bewick's swan, common gull, and guillemot. Dabbling ducks were more frequently infected than other ducks and Anseriformes; this distinction was probably related to bird behavior rather than population sizes. Waders did not appear to play a role in the epidemiology of avian influenza in Europe, in contrast to the Americas. The high virus prevalence in ducks in Europe in spring as compared with North America could explain the differences in virus–host ecology between these continents. Most influenza A virus subtypes were detected in ducks, but H13 and H16 subtypes were detected primarily in gulls. Viruses of subtype H6 were more promiscuous in host range than other subtypes. Temporal and spatial variation in influenza virus prevalence in wild birds was observed, with influenza A virus prevalence varying by sampling location; this is probably related to migration patterns from northeast to southwest and a higher prevalence farther north along the flyways. We discuss the ecology and epidemiology of avian influenza A virus in wild birds in relation to host ecology and compare our results with published studies. These data are useful for designing new surveillance programs and are particularly relevant due to increased interest in avian influenza in wild birds.

## Author Summary

Significant gaps in our knowledge of the ecology of avian influenza in wild migratory birds have become apparent during recent outbreaks of H5N1 highly pathogenic avian influenza, in particular in relation to the risk of virus spread by wild birds. An eight-year surveillance study, which included more than 36,000 wild birds tested for low pathogenic avian influenza, provides new information on host species, prevalence, and temporal and geographical variation of avian influenza in wild migratory birds in Europe. Dabbling ducks harbored nearly all known influenza virus subtypes, with the exception of H13 and H16, which were found primarily in gulls. In contrast to American studies, waders did not play a role in the epidemiology of avian influenza in Europe. This study provides important information on the ecology and epidemiology of avian influenza A virus and could assist in the design of new surveillance studies for high and low pathogenic avian influenza in wild birds.

## Introduction

Birds of wetlands and aquatic environments such as the Anseriformes (particularly ducks, geese, and swans) and Charadriiformes (particularly gulls, terns, and shorebirds) are thought to constitute the major natural reservoir for avian influenza A virus [1,2]. Influenza A viruses of all hemagglutinin (HA) and neuraminidase (NA) subtypes (H1–H16 and N1–N9) and most HA/NA combinations have been identified in the wild bird reservoir [3,4]. Anseriformes and Charadriiformes are distributed globally, except for the most arid regions of the world, and represent an almost global coverage of influenza A virus host species [1,2]. In birds, influenza viruses preferentially infect cells lining the intestinal tract and are excreted in high concentrations in their feces. Transmission is thought to be achieved primarily via the fecal–oral route [1], which likely represents an efficient way to transmit viruses between waterfowl, by shedding the virus via feces into the surface water [1].

The prevalence of avian influenza A viruses in their natural hosts depends on geographical location, seasonality, and species. For instance, the prevalence of avian influenza A viruses in ducks in North America varies from less than 1% during spring migration to 30% prior to and during fall migration, but large variations in virus prevalence have been observed in different surveillance studies [1,4,5]. The peak in prevalence during fall migration is believed to be related to the large number of young immunologically naïve birds of that breeding season [1,2,6,7]. Although extensive data exist on surveillance studies of influenza A viruses in ducks and shorebirds in North America [4,5], limited up-to-date information is available for Eurasia, Africa, South America, and Oceania, and only for limited numbers of species [8–11]. Because of the apparent species-specific niches of certain HA subtypes such as H13 and the recently discovered H16 [3,12–14], as yet unidentified influenza A viruses may exist in nature. Information about influenza A viruses in Eurasia and North America is of particular interest because the influenza A viruses found in Eurasian wild birds are genetically distinct from those of wild birds in the Americas [1,2]. The direct zoonotic potential of several Eurasian lineage avian influenza A viruses is currently the cause of serious concern [15–18]. The increasing problems with outbreaks of highly pathogenic avian influenza (HPAI), the potential spread of HPAI H5N1 by wild birds over large geographic areas, and the threat certain avian influenza A viruses pose to public and animal health emphasize the need for more information on the ecology of avian influenza A viruses circulating in the wild bird reservoir. Our current knowledge of the epidemiology of avian influenza A viruses, virus ecology in relation to host ecology, the temporal and spatial patterns of avian influenza A viruses in their natural hosts, the role of potential new hosts in the influenza A virus ecology, and the interaction between wild birds and poultry are still very limited.

Traditionally, influenza A virus surveillance studies in wild birds have been performed by direct virus isolation from fecal samples or cloacal swabs in embryonated hen's eggs [19]. This method is labor intensive due to the handling time of each of the individual samples, and is quite sensitive to laboratory contaminations, in particular if blind passage is used routinely during virus isolation attempts. Currently this diagnostic method is being replaced in many laboratories by molecular diagnostic tests, such as conventional or real-time RT-PCR methods targeting highly conserved gene segments of the influenza A virus. Such molecular methods allow the rapid identification of influenza A virus positive specimens from large collections of samples, which can then be used for targeted virus isolation attempts [20–22]. In this study, we present data on the prevalence of influenza A viruses from our ongoing wild bird surveillance studies. From 1998 to 2006, we screened more than 36,000 samples collected from 323 bird species using molecular diagnostics. The sample collection includes many bird species reported to be permissive to avian influenza A virus in earlier influenza A virus surveillance studies [1,11]. To obtain more detailed information on potential host species, large numbers of samples of birds from different bird families and geographical locations were included. This was done in part because earlier studies relied solely on virus isolation in embryonated hen's eggs as a screening method for investigating whether molecular detection methods would yield different results. Of all samples, 90% were from The Netherlands and Sweden, 4.5% from elsewhere in Northern Europe (seven countries, multiple sites), and 5.5% from other parts of the world, including Africa (Nigeria, Ghana), North America (United States, Canada), South America (Argentina), Asia (Kazakhstan, South Korea), the Arctic (Norway, Iceland), and the Antarctic Peninsula. All samples were taken from healthy birds. We used this data to describe temporal and spatial patterns in influenza A virus prevalence in different wild migratory bird species.

## Results

### Avian Influenza A Virus Surveillance in Wild Birds

From 1998 to 2006 we sampled 36,809 birds belonging to 323 species of 18 orders (Table 1). Lists of species, sample numbers, and locations are included in Tables S1 and S2. All influenza A virus positive bird species were obtained in Northern Europe, unless mentioned otherwise. Of the 992 RT-PCR positive samples, 332 virus isolates were recovered, yielding an overall recovery rate of 33.5%. The majority of the samples from which we were unable to isolate virus had threshold cycle values above 35, which indicates a low viral load. In addition, a small subset of the samples was initially stored at −20 °C, which may have a negative effect on the virus isolation rate. All influenza A virus isolates were obtained from birds belonging to the orders of Anseriformes and Charadriiformes migrating along the East Atlantic flyway [1,2].

**Table 1:**
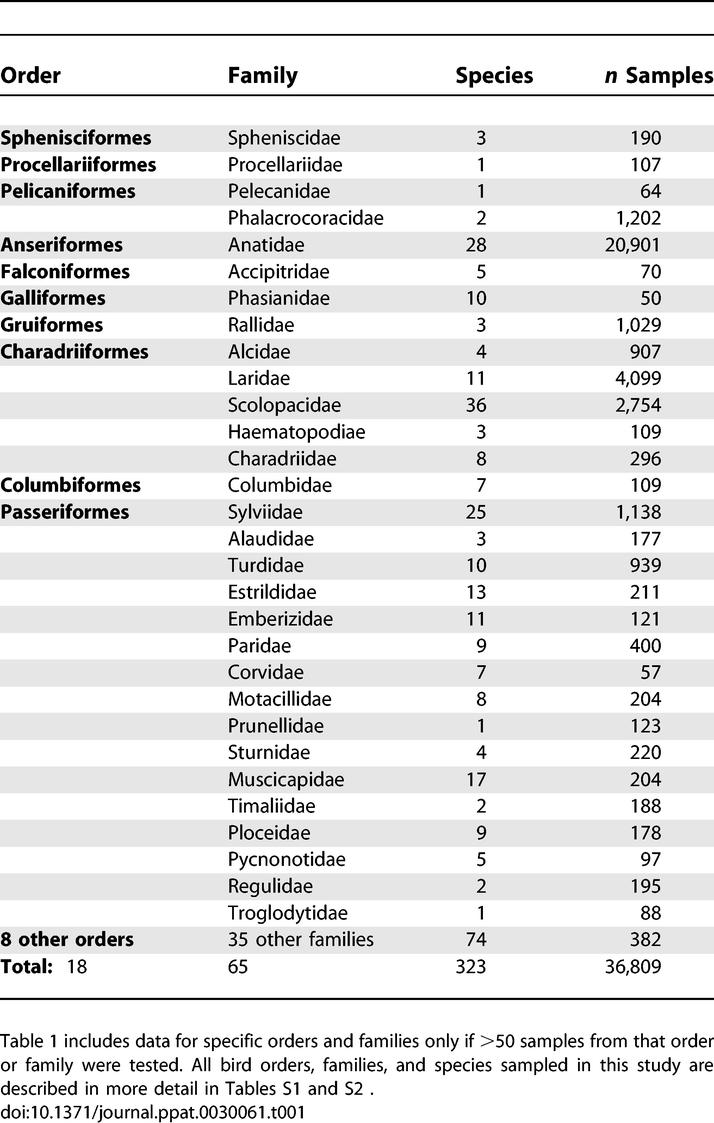
Overview of the Bird Samples Analyzed in This Study

### Prevalence of Influenza A Viruses in Anseriformes

**Figure 1:**
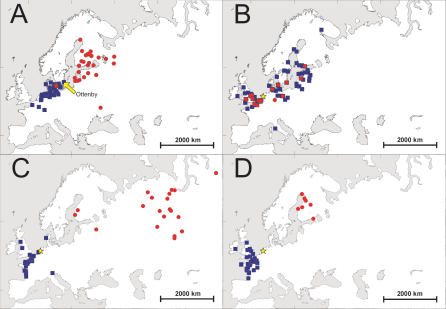
Summer and Winter Distribution of Different Duck Species Ringed in The Netherlands and Sweden (A) Recoveries of mallards *(Anas platyrhynchos)* ringed at Ottenby Bird Observatory, southeast Sweden (yellow arrow), and found in 2002 and 2003 in the period May–August (*n* = 29, red circles) and November–February (*n* = 54, blue squares). (B) Ringing sites of mallards *(Anas platyrhynchos)* found in The Netherlands (yellow star) in 1976–2005 in the periods May–August (*n* = 61, red circles) and November–February (*n* = 311, blue squares). (C) Recoveries of wigeons *(Anas penelope)* ringed in The Netherlands (yellow star) and found in 1998–2005 in the periods May–August (*n* = 20, red circles) and November–February (*n* = 38, blue squares). (D) Recoveries of teals *(Anas crecca)* ringed in the Netherlands (yellow star) and found in 1998–2005 in the periods May–August (*n* = 7, red circles) and November–February (*n* = 36, blue squares) [40]. The blue squares represent winter recoveries (November–February) and red circles represent summer recoveries (May–August).

The prevalence of influenza A virus in the different duck, goose, and swan species is presented in Table 2. The prevalence in dabbling ducks was 6.1%. Mallards and teals had a higher prevalence of the virus than wigeons, pintails, gadwalls, and shovelers combined (7.2% versus 3.0%, Pearson X2-test, *p* < 0.001). The sampled dabbling ducks all migrate along the East Atlantic flyway and were sampled during fall migration (Sweden) or upon arrival and stay at their wintering grounds (The Netherlands) (Table S1). Figure 1 shows the ring recovery for mallards ringed at Ottenby Bird Observatory (Öland, Sweden) in 2002 and 2003, and mallards, Eurasian wigeons, and common teals ringed in The Netherlands from 1998 to 2005.

**Table 2:**
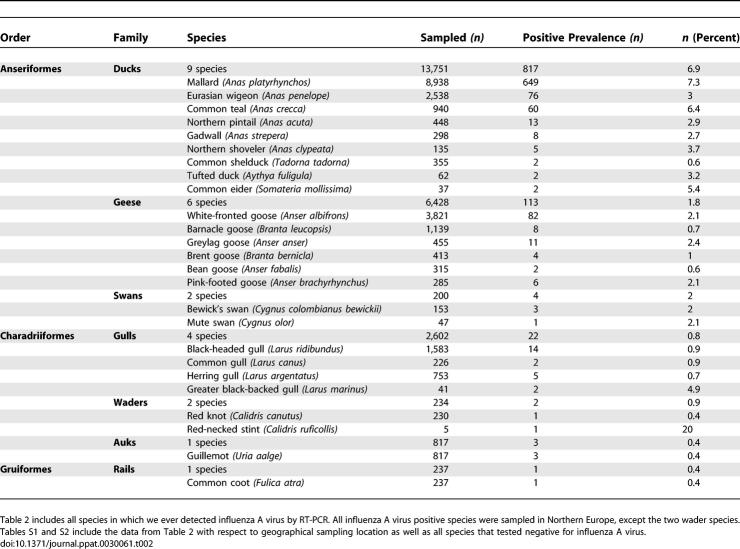
Prevalence of Influenza A Virus in Wild Birds Sampled in This Study

**Figure 2:**
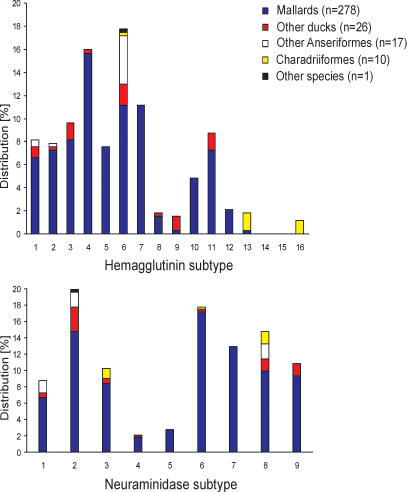
Distribution of HA and NA Subtypes in Influenza A Virus Isolates Obtained from Wild Birds Data from all 332 virus isolates were included with the distribution of the HA subtypes shown in the top panel and the NA subtypes in the bottom panel.

Influenza A viruses were occasionally detected in common eiders, common shelducks, and tufted ducks, which belong to the guilds of stifftails, shelducks, and pochards, respectively. Influenza A viruses were not detected in 20 other ducks belonging to eight additional species. Influenza A viruses of subtypes H1–H13 were obtained from mallards; H1, H4, H6, and H9 from Eurasian wigeons; H1, H3, H6, and H8 from common teals; H9 from gadwalls; H2 from northern pintails; and H11 from northern shovelers (Figure 2). The HA subtype distribution in mallards was different from that in all other ducks (Pearson χ2-test, *p* < 0.001). Note, however, that relatively few virus isolates were obtained from other duck species (*n* = 26).

Samples were obtained from eight goose and three swan species (Tables 2 and S2). Influenza A viruses were detected in white-fronted, barnacle, greylag, brent, bean, and pink-footed geese, as well as bewick's and mute swans. HA subtypes detected in geese and swans were H1 (9.5%), H2 (4.8%), H6 (81%), and H9 (4.8%). Thus, the vast majority of influenza A virus isolates obtained from geese and swans were of the H6 subtype: H6N1 (18%), H6N2 (35%), and H6N8 (47%).

### Prevalence of Influenza A Viruses in Charadriiformes

Within the Laridae family, a total of 4,099 samples were obtained from nine gull and two tern species that originated predominantly from Northern Europe (Tables 1, S1, and S2). Influenza A viruses were detected in black-headed, common, herring, and greater black-backed gulls (Table 2), but not in five other gull species and two tern species (Table S2). Virus prevalence varied greatly with respect to colonies sampled, timing, and geography. Prevalence of 60% was detected in juvenile black-headed gulls during fall migration in Öland, Sweden [3], while the overall prevalence was only 0.8% (Table 2). Influenza A virus was undetectable in many different colonies during breeding season over multiple years in The Netherlands and Sweden. Positive samples were predominantly obtained in June, July, and August. Influenza A virus subtypes isolated from gulls were H6N8 (10%), H13N6 (10%), H13N8 (40%), and H16N3 (40%).

We obtained 3,159 samples from 47 wader species in a variety of sampling sites in Europe, 60% of which were taken during fall migration, 35% during spring migration, and 5% at the breeding grounds. We obtained one positive sample from a red knot out of 230 birds caught at Delaware Bay, United States, in early May 2005 and one from a red-necked stint out of five sampled in South Korea (Table 2). All other waders were negative for influenza A virus.

Within the Alcidae family, we obtained 907 samples from four bird species. Three influenza A virus positive samples were obtained from 817 guillemots; all were H6N2 viruses [23].

### Prevalence of Influenza A Viruses in Other Bird Species

An influenza A virus was detected in one out of 237 common coots sampled. More than 10,000 samples were collected from wild birds in 15 orders other than the Anseriformes, Charadriiformes, and Gruiformes, but no influenza A viruses were detected in those samples (Table S2).

### Temporal and Longitudinal Variation in Prevalence of Influenza A Viruses in Mallards

**Figure 3:**
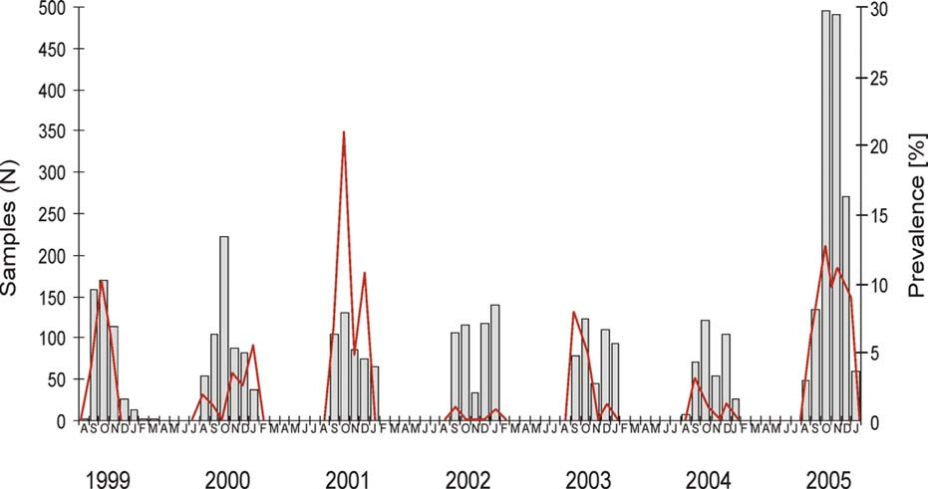
Annual Influenza A Virus Prevalence in Mallards during Fall Migration in The Netherlands from 1999 to 2005 Bars indicate the number of samples collected per month (left *y*-axis), and the red line indicates the number of samples positive for influenza A virus by RT-PCR (right *y*-axis).

For mallards, we compared the prevalence of influenza A virus from 1999 to 2005 in The Netherlands. The sample size was approximately evenly distributed from 1999 to 2004, but increased in 2005 in response to the HPAI H5N1 threat. The peak prevalence varied from 0.93% in September 2002 to 20.76% in October 2001. The peak prevalence for most years was in September and October, with the exception of 2000 (January; see Figure 3). Similar fluctuations in peak prevalence were observed in Eurasian wigeons (0.83% in December 2002; 20% in September 2005) and common teals (4% in November 2000; 30% in November 2005) (unpublished data).

**Figure 4:**
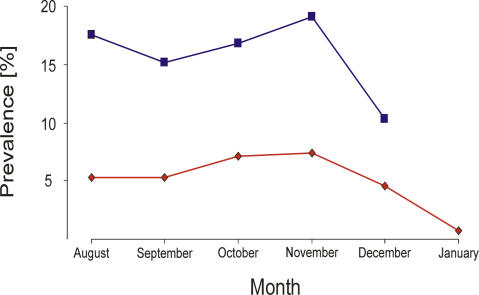
Trend Lines for Influenza A Virus Prevalence in Mallards Caught in Sweden and The Netherlands during Fall Migration The blue line and filled squares (▪) represent the proportion (%) of influenza A virus positive mallards caught and sampled in Sweden between 2002 and 2005 at Ottenby Bird Observatory. The red line and filled diamonds (♦) represent mallards caught at various locations in The Netherlands from 1998 to 2005.

Although sample size may vary somewhat between years and peak prevalence may vary considerably, we calculated a generalized trend line for influenza A virus in mallards in The Netherlands and Sweden. The winter and summer distribution of these mallard populations is shown in Figure 1. Virus prevalence in mallards in Sweden was ∼3-fold higher as compared with The Netherlands (Figure 4). For both countries, influenza A virus prevalence was already high upon arrival on the sampling sites in August, only to drop after November.

### Population Demography and Prevalence of Influenza A Viruses in Dabbling Ducks

The prevalence of influenza A viruses with respect to age and sex was determined for mallards and Eurasian wigeons. The prevalence was different between juveniles (year 1) and adults (consecutive years). Influenza A virus prevalence was 6.8% for juvenile ducks (*n* = 2038) and 2.8% for adults (*n* = 895). Juvenile ducks thus had a greater chance to be influenza A virus positive than adults (RR: 2.24, 95% CI: 1.61 to 3.71). No apparent differences in prevalence were observed between male (*n* = 4737) and female (*n* = 3114) ducks (RR: 1.13, 95% CI: 0.944 to 1.35).

### Prevalence of HA Subtypes and HA/NA Subtype Combinations in Wild Birds

H6 (17.8%) and H4 (16%) were the most abundantly detected HA subtypes, followed by H7 (11.1%), H3 (9.6%), H11 (8.7%), H1 (8.1%), H2 (7.8%), H5 (7.5%), H10 (4.8%), H12 (2.1%), H8 (1.8%), H13 (1.8%), H9 (1.5%), and H16 (1.2%). H14 and H15 were never detected. Viruses of the H13 and H16 subtypes were primarily obtained from Charadriiformes (Figure 2). Viruses of subtype H6 were obtained relatively frequently from wild birds other than mallards. All H5 and H7 isolates were low pathogenic avian influenza viruses [24].

The most frequently detected NA subtype was N2 (19.9%), followed by N6 (17.8%), N8 (14.8%), N7 (13%), N9 (10.8%), N3 (10.2%), N1 (8.7%), N5 (2.7%), and N4 (2.1%) (Table 3). Subtypes N5 and N7 were only found in viruses isolated from mallards. From Charadriiformes we only obtained viruses of the N3, N6, and N8 subtypes and from geese and swans only of the N1, N2, and N8 subtypes (Figure 2).

**Table 3:**
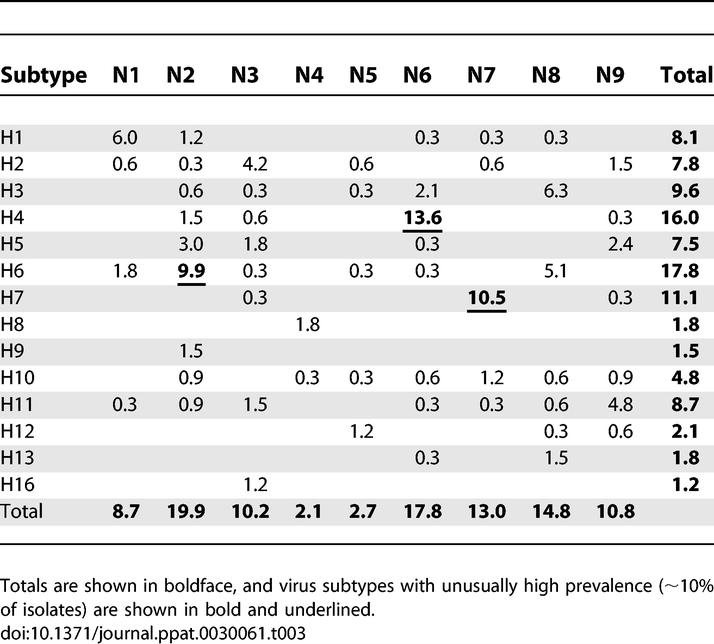
Proportion of Influenza A Virus HA and NA Subtypes among 332 Virus Isolates

In total, 55 different HA/NA subtype combinations were detected (Table 3). The most frequently detected subtype combination was H4N6, comprising 13.6% of all isolated influenza A viruses, followed by H7N7 (10.5%) and H6N2 (9.9%). Viruses containing H8 matched only with N4 and viruses containing H16 only with N3 (Table 3).

## Discussion

Recent improvements in molecular diagnostic tests have facilitated high-throughput screening of wild birds for influenza A virus. Despite the introduction of the molecular tests and the wide range of bird species tested, only a few “new” influenza A virus hosts were identified: barnacle, bean, brent, and pink-footed goose, bewick's swan, common gull, and guillemot. It is thus reassuring that the use of classical methods for virus detection in numerous surveillance studies has not resulted in an apparent biased detection toward viruses that can be isolated easily in embryonated hens' eggs; therefore, it remains a viable approach for virus detection. However, in our hands, virus detection by RT-PCR was more sensitive than using classical tests, since viruses were isolated from only one-third of the RT-PCR positive samples. Even under ideal conditions of transport, storage, and processing, not all RT-PCR positive samples yielded virus isolates.

We confirmed the high virus prevalence of dabbling ducks in fall as observed in previous studies in the Northern Hemisphere [1]. Our data indicate that timing relative to migration, instead of the absolute time point, is a determinant of virus prevalence. High virus prevalence early in fall migration likely declines gradually as the migration proceeds, thus forming a north–south gradient of virus prevalence even within single species. This explains prevalence differences in earlier surveillance studies [4,5]. Influenza A virus prevalence was generally higher in juvenile ducks as compared with adults, as reported for North America [5–7]. The estimated yearly turnover of mallards in Northern Europe is roughly one-third; 56% of the juvenile mallards die during their first year and the mortality in adult birds is ∼40% [24]. Thus, one-third of the mallard population consists of juvenile birds, which are immunologically naïve and therefore probably more susceptible to influenza A virus[1,6].

**Figure 5:**
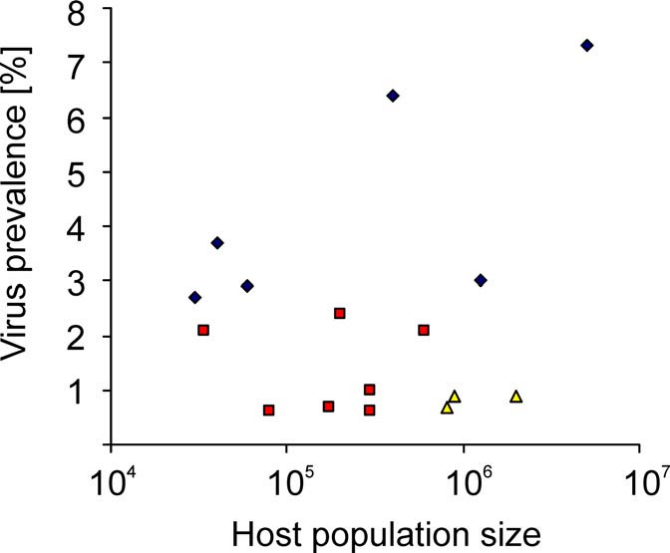
Relation between Influenza A Virus Prevalence in Avian Hosts and Their Population Sizes Species were categorized in dabbling ducks (blue diamonds; mallard [population 5,000,000; prevalence 7.3%], Eurasian wigeon [1,250,000; 3%], common teal [400,000; 6.4%], northern pintail [60,000; 2.9%], gadwall [30,000; 2.7%], and northern shoveler [40,000; 3.7%]); geese (red squares; white-fronted goose [600,000; 2.1%], barnacle goose [176,000; 0.7%], greylag goose [200,000; 2.4%], brent goose [300,000; 1%], bean goose [80,000; 0.6%], and pink-footed goose [34,000; 2.1%]); and gulls (yellow triangles; black-headed gull [2,000,000, 0.9%], common gull [500,000, 0.9%], and herring gull [800,000, 0.7%]). Virus prevalence in these species was plotted against their respective population sizes [26]. Species are included if >200 samples were tested for influenza A virus. There was no correlation between influenza A virus prevalence and population size, but there was a clustering of data points according to the species categories.

The influenza A virus prevalence in mallards was comparable to that of other dabbling ducks. Viruses were also detected in ducks belonging to other guilds, but the prevalence was lower. Influenza A virus was detected in 811 of 13,297 dabbling ducks, but in only six of 440 other ducks (Pearson χ^2^-test, *p* = < 0.001). Analysis of 7,130 samples from 11 goose and swan species revealed that virus prevalence was also low, ranging from 0.7% to 2.4%. Several factors could contribute to the high virus prevalence in dabbling ducks as compared with other species. The dabbling behavior itself is likely an important factor; virus excreted in surface waters via feces may efficiently transmit viruses to other ducks that feed on the same waters. Influenza A virus can remain infectious for prolonged periods in surface water depending on temperature, salinity, and pH [25]. The prolonged presence of influenza A viruses in surface water may enable the spread of viruses in different host sub-populations that otherwise would be separated in time and space. In contrast, diving ducks forage deeper under the surface and more often in marine habitats, and most goose and swan species graze in pastures and agricultural fields. Such differences in feeding behavior could lead to less-efficient virus transmission and thus account for the differences in prevalence. Population size and age structure could be additional important factors enabling the annual co-circulation of multiple virus subtypes within the same (meta-) populations [26]. The dabbling duck populations are estimated at between 5,000,000 and 10,000,000 in Northern Europe alone [27]. Mallards are the most abundant species, followed by Eurasian wigeon and common teal [27]. We observed the highest virus prevalence in two of these species: mallards and common teals. The population estimates for goose species in Northern Europe are significantly lower as compared with the dabbling ducks [27]. The smaller population sizes could limit in general the potential of perpetuation of influenza A virus in these species and in particular the continuous co-circulation of multiple virus subtypes. When we plotted the influenza A virus prevalence in duck, goose, and gull species as a function of population size (Figure 5), population size did not appear to be the main correlate of virus prevalence (R^2^ = 0.0001). The relative clustering of the data points from the duck, goose, and gull species (Figure 5) suggests that other factors (taxonomy, behavior, etc.) could determine virus prevalence.

The population size of 2,000,000 black-headed gulls in Northern Europe seems to be sufficiently large for the continuous circulation of influenza A viruses. Behavioral factors influencing influenza A virus ecology in gulls could include colony breeding, gregariousness during migration and wintering, feeding patterns, and the mixing of different populations of birds. From our data, it appears that virus prevalence in gulls peaks shortly after they have left their breeding grounds.

Surveillance studies performed along the East Coast of North America suggested a distinct role for wader species in the perpetuation and maintenance of certain influenza A virus subtypes [4,12]. It was suggested that different families of wetland birds are involved in perpetuating influenza viruses and that waders may carry the virus north to the duck breeding grounds in spring. We detected influenza A virus in one shorebird sample obtained from Delaware Bay (United States) and one from South Korea. The differences in prevalence between our American wader data and those described by others [4,12] could be due to sampled species, sampling procedures, and timing. Within our European surveillance study, not a single influenza A virus was detected in waders. Although the majority of our wader samples were collected during fall migration, a reasonable sample size was collected during spring migration. Thus, there is no evidence that waders play a role in the perpetuation of influenza A virus in Europe. The recently intensified surveillance in waders, including serological data collection, may allow a definitive conclusion about their role in the influenza virus ecology in Europe.

Although historically influenza A viruses have been obtained from more than 105 species of 26 different families [2], we did not detect significant influenza virus prevalence in species other than those belonging to the orders Anseriformes and Charadriiformes. We therefore suggest that although multiple bird species can be infected, their contribution to the overall virus ecology could be limited; infections of these hosts, although potentially with high peak prevalence, may be only transient.

Influenza A virus subtypes H1–H12 were isolated frequently from mallards, and several of these subtypes were also detected in Eurasian wigeons, common teals, gadwalls, and northern shovelers. The absence of subtypes H14 and H15 in our collection was probably due to the geographical separation of virus hosts [28,29]. Because all HA subtypes isolated from Anseriformes were also isolated from mallards, it is likely that mallards play a pivotal role in the perpetuation of influenza A virus subtypes H1–H12 in Europe. Subtypes H5 and H7 were rarely detected in longitudinal studies of ducks in Canada, whereas in this European study, they represent 7.5% and 11.1% of viruses obtained from ducks. The most common virus subtypes in Europe—H3, H4, H6—were also common subtypes in Canada.

The predominant isolation of H13 and H16 viruses from gull species confirms the common notion that these viruses belong to the influenza A virus “gull lineage.” H13 and H16 viruses are genetically distinct from viruses from other hosts and seem to have adapted to replication in gull hosts in particular [3,12].

Interestingly, viruses of the H6 subtype seem to have a broader host range compared with that of other virus subtypes in our study. Of the influenza viruses obtained from birds other than dabbling ducks and gulls, 79% were H6 viruses. H6 viruses were isolated from gulls, auks, swans, and geese. The relative abundance of this subtype in ducks does not explain the large variety of species from which these viruses were isolated; H4 and H7 viruses were also detected frequently in ducks, but rarely in other birds. H6 viruses have been transmitted from wild birds to poultry on several occasions [30,31], providing further evidence for the ability of these viruses to be transmitted between different bird species.

HPAI H5N1 viruses have caused large-scale outbreaks in poultry in Southeast Asia since 1997 and have also been transmitted to a variety of mammalian species, including humans [17,18,32] . Until 2005, wild migratory birds probably did not play a significant role in the epidemiology and spread of HPAI H5N1, although the virus was detected sporadically in wild birds. A large-scale outbreak in wild migratory birds occurred in April–June 2005 at Lake Qinghai in China [33–35], after which the HPAI H5N1 virus rapidly spread westward across Asia, Europe, the Middle East, and Africa. Since then, affected wild birds have been reported in several countries [36], but even in areas with significant outbreaks in poultry, the virus prevalence in wild birds is low and their role in spreading the disease is unclear. It is likely that the influenza A surveillance studies in wild birds such as those presented here could provide “early warning” signals for the introduction of HPAI H5N1 into new regions [37]. The current increased interest in influenza virus surveillance in wild and domestic birds provides a unique opportunity to increase our understanding not only of HPAI epidemiology but also of the ecology of low pathogenic avian influenza viruses in their natural hosts.

## Materials and Methods

### Specimens.

Birds were trapped by expert ornithologists using duck decoys, duck traps, wader funnel traps, mist nets, clap nets, cannon nets, or Helgoland traps. Cloacal swabs were collected using sterile cotton swabs of two different sizes depending on the size of the birds. The cloacal swabs were stored in transport medium (Hank's balanced salt solution containing 0.5% lactalbumin, 10% glycerol, 200 U/ml penicillin, 200 μg/ml strepromycin, 100 U/ml polymyxin B sulfate, 250 μg/ml gentamycin, and 50 U/ml nystatin [ICN Pharmaceuticals, http://www.icnpharm.com]) and shipped to the laboratory where they were stored at −80 °C upon analysis. Before shipment, the samples were stored at 4 °C for less than a week, at −80 °C if such freezers were available nearby the sampling site, and at −20 °C only if rapid transport or storage at −80 °C was practically impossible.

### Sample location.

Samples were obtained from 323 different bird species belonging to 18 different orders and a wide variety of sampling locations. The majority of samples were obtained consistently from the same sites in The Netherlands (Krimpen a/d Lek, 51°54′N 4°41′E; and Lekkerkerk, 51°54′N 4°38′E) and Sweden (Ottenby Bird Observatory, Öland, 56°12′N 16°24′E). Samples were also collected during short-term sampling expeditions at different sites in Europe, Asia, Africa, North America, South America, Antarctic, and the Arctic. In 2005, numerous sampling sites were added in response to potential HPAI H5N1 threats.

### RNA isolation and virus detection.

RNA isolation and RT-PCR were performed as described previously for samples obtained until 2002 [22]. From 2003 onward, RNA was isolated using a MagnaPure LC system with the MagnaPure LC Total nucleic acid isolation kit (Roche Diagnostics, http://www.roche-diagnostics.nl) and influenza A virus was detected using a real-time RT-PCR assay targeting the matrix gene [21]. To ensure efficient influenza A virus detection, the published probe sequence was changed to 6-FAM-TTT-GTG-TTC-ACG-CTC-ACC-GTG-CC-TAMRA-3′**,** based on the avian influenza A virus sequences available from public databases. Amplification and detection was performed on an ABI7700 with the TaqMan EZ RT-PCR Core Reagents kit (Applied Biosystems, http://www.appliedbiosystems.com) using 20 μl of eluate in an end volume of 50 μl. Pools of five individual samples were prepared and processed in parallel with several negative (three negative controls on 32 samples) and positive (one positive control on 32 samples) control samples in each run. Upon identification of influenza A virus positive pools, the RNA isolation and RT-PCR procedures were repeated for the individual samples within each positive pool (again processed in parallel with three negative controls and one positive control per 32 samples), and individual RT-PCR positive samples were subsequently used for virus isolation. RNA isolation and real-time RT-PCR were performed by the diagnostic facility of the Erasmus MC Department of Virology.

### Virus isolation and characterization.

For influenza A virus RT-PCR positive samples, 200 μl of the original material was inoculated into the allantoic cavity of 11-d-old embryonated hens' eggs. The allantoic fluid was harvested 2 d after inoculation and influenza A virus was detected using hemagglutination assays with turkey erythrocytes. When no influenza A virus was detected upon the initial virus isolation attempt, the allantoic fluid was passaged once more in embryonated hens' eggs. The HA subtype of virus isolates were characterized using a hemagglutination inhibition assay with turkey erythrocytes and subtype-specific hyperimmune rabbit antisera raised against all HA subtypes [3].

### Sequence analysis.

The NA subtype of virus isolates was characterized by RT-PCR and sequencing. RT-PCR was performed using primers specific for the conserved non-coding regions of NA, essentially as described by others [38]. PCR products were purified from agarose gels using the Qiaquick Gel Extraction kit (Qiagen, http://www.qiagen.com) and sequenced. Sequencing was performed using the Big Dye terminator sequencing kit version 3.0 (Amersham Pharmacia Biotech, http://www.gelifesciences.com) and a 3100 genetic analyzer (Applied Biosystems), according to the instructions of the manufacturer. Nucleotide sequences were aligned using the Clustal W program running within the BioEdit software package, version 5.0.9. NA nucleotide sequences were analyzed with the basic local alignment search tool available from GenBank [39] to identify the NA subtype.

### Statistics.

The 95% confidence interval analysis and the Pearson χ2-test were used for analysis of the dataset used in this study.

## Supporting Information

Table S1 Bird Species that Tested Positive for Influenza A Virus in This Study Table S1 includes data on all species in which influenza A virus was detected by RT-PCR, including geographical sampling location and sample size.
(48 KB DOC)

Table S2 Bird Species That Tested Negative for Influenza A Virus in This Study Table S2 includes data on all species in which no influenza A virus was detected by RT-PCR, including geographical sampling location and sample size.
(435 KB DOC)

## Abbreviations

HA: hemagglutinin
HPAI: highly pathogenic avian influenza
NA: neuraminidase
