# Supplementary material for: Spatial, Temporal, and Species Variation in Prevalence of Influenza A Viruses in Wild Migratory Birds
Source: PLoS Pathog. 2007 May 11;3(5):e61. doi: 10.1371/journal.ppat.0030061 (PMC1876497; doi:10.1371/journal.ppat.0030061)
Supplement: Table S1 — includes data on all species in which influenza A virus was detected by RT-PCR, including geographical sampling location and sample size. (48 KB DOC) [file ppat.0030061.st001.doc]

Table S1: Bird species that tested positive for influenza A virus in this study. This table includes data on all species in which influenza A virus was detected by RT-PCR, including geographical sampling location and sample size.

|  | Family | Species | Sampled | % | Sampled  Netherlands | % | Sampled  Sweden | % | Sampled  Other | % |
| --- | --- | --- | --- | --- | --- | --- | --- | --- | --- | --- |
| Anseriformes | Anatidae | Mallard (*Anas platyrhynchos*) | 8938 | 7.3 | 5325 | 5.3 | 3613 | 10.1 |  |  |
|  |  | Eurasian Wigeon (*Anas penelope*) | 2538 | 3 | 2519 | 3.0 | 19 | 5.3 |  |  |
|  |  | Common Teal (*Anas crecca*) | 940 | 6.4 | 856 | 5.8 | 84 | 11.9 |  |  |
|  |  | Northern Pintail (*Anas acuta*) | 448 | 2.9 | 431 | 2.8 | 17 | 5.9 |  |  |
|  |  | Gadwall (*Anas strepera*) | 298 | 2.7 | 298 | 2.7 |  |  |  |  |
|  |  | Northern Shoveler (*Anas clypeata*) | 135 | 3.7 | 134 | 3.7 | 1 | 0 |  |  |
|  |  | Common Shelduck (*Tadorna tadorna*) | 355 | 0.6 | 64 | 0 | 291 | 0.6 |  |  |
|  |  | Tufted Duck (*Aythya fuligula*) | 62 | 3.2 | 52 | 3.8 | 18 | 0 |  |  |
|  |  | Common Eider (*Somateria mollissima*) | 37 | 5.4 | 10 | 20 | 22 | 0 | 5^1^ | 0 |
|  |  | White-fronted Goose (*Anser albifrons*) | 3821 | 2.1 | 3821 | 2.1 |  |  |  |  |
|  |  | Barnacle Goose (*Branta leucopsis*) | 1139 | 0.7 | 811 | 1.0 | 328 | 0 |  |  |
|  |  | Greylag Goose (*Anser anser*) | 455 | 2.4 | 455 | 2.4 |  |  |  |  |
|  |  | Brent Goose (*Branta bernicla*) | 413 | 1.0 | 401 | 1.0 | 12 | 0 |  |  |
|  |  | Bean Goose (*Anser fabalis*) | 315 | 0.6 | 315 | 0.6 |  |  |  |  |
|  |  | Pink-footed Goose (*Anser brachyrhynchus*) | 285 | 2.1 | 285 | 2.1 |  |  |  |  |
|  |  | Bewick's Swan (*Cygnus colombianus bewickii*) | 153 | 2 | 153 | 2 |  |  |  |  |
|  |  | Mute Swan (*Cygnus olor*) | 47 | 2.1 | 37 | 2.7 | 10 | 0 |  |  |
| Charadriiformes | Laridae | Black-headed Gull (*Larus ridibundus*) | 1583 | 0.9 | 684 | 0.73 | 429 | 9 | 470^2^ | 0 |
|  |  | Common Gull (*Larus canus*) | 226 | 0.9 | 224 | 0.9 | 2 | 0 |  |  |
|  |  | Herring Gull (*Larus argentatus*) | 753 | 0.7 | 735 | 0.7 | 16 | 0 | 2^1^ | 0 |
|  |  | Greater Black-backed Gull (*Larus marinus*) | 41 | 4.9 | 26 | 3.8 | 14 | 7.1 |  |  |
|  | Scolopacidae | Red Knot (*Calidris canutus*) | 230 | 0.4 | 90 | 0 |  |  | 133^4^ | 0.8 |
|  |  | Red-necked Stint (*Calidris ruficollis*) | 5 | 20 |  |  |  |  | 5^5^ | 20 |
|  | Alcidae | Guillemot (*Uria aalge*) | 817 | 0.4 | 6 | 0 | 623 | 0.5 | 88^3^ | 0 |
| Gruiformes | Rallidae | Common Coot (*Fulica atra*) | 237 | 0.4 | 235 | 0.4 | 2 | 0 |  |  |

^1^ Sampled in Iceland

^2^ Sampled in Estonia, Iceland, Latvia and Finland

^3^ Sampled in Norway

^4^ Sampled in Delaware bay, USA

^5^ Sampled in South Korea
