# Supplementary material for: Spatial, Temporal, and Species Variation in Prevalence of Influenza A Viruses in Wild Migratory Birds
Source: PLoS Pathog. 2007 May 11;3(5):e61. doi: 10.1371/journal.ppat.0030061 (PMC1876497; doi:10.1371/journal.ppat.0030061)
Supplement: Table S2 — includes data on all species in which no influenza A virus was detected by RT-PCR, including geographical sampling location and sample size. (435 KB DOC) [file ppat.0030061.st002.doc]

Table S2: Bird species that tested negative for influenza A virus in this study. This table includes data on all species in which no influenza A virus was detected by RT-PCR, with respect to geographical sampling location and sample size. The following abbreviations are used; AF = Africa, ANT = Antarctica, AS = Asia, EU = Europe, NA = North America and SA = South America.

| **Order** | **Family** | **Species (English)** | ***Species (Latin)*** | **N sampled** | **Geographical Region** |
| --- | --- | --- | --- | --- | --- |
| Sphenisciformes | Spheniscidae | Adelie Penguin | *Pygoscelis adeliae* | 30 | ANT |
|  |  | Chinstrap Penguin | *Pygoscelis antartica* | 29 | ANT |
|  |  | Gentoo Penguin | *Pygoscelis papua* | 131 | ANT |
|  |  |  |  |  |  |
| Podicipediformes | Podicipedidae | Great Crested Grebe | *Podiceps cristatus* | 1 | EU |
|  |  | Little Grebe | *Tachybaptus ruficollis* | 1 | EU |
|  |  |  |  |  |  |
| Procellariiformes | Procellariidae | Fulmar | *Fulmarus glacialis* | 107 | EU |
|  |  |  |  |  |  |
| Pelicaniformes | Pelecanidae | American White Pelican | *Pelecanus erythrorhynchos* | 64 | NA |
|  |  |  |  |  |  |
|  | Phalacrocoracidae | Antarctic Shag | *Phalacrocorax bransfieldensis* | 46 | ANT |
|  |  | Great Cormorant | *Phalacrocorax carbo* | 1156 | EU, NA |
|  |  |  |  |  |  |
| Ciconiiformess | Ardeidae | Black-crowned Night-Heron | *Nycticorax nycticorax* | 2 | AS |
|  |  | Chinese Little Bittern | *Ixobrychus sinensis* | 3 | AS |
|  |  | Grey Heron | *Ardea cinerea* | 18 | AS, EU |
|  |  | Little Bittern | *Ixobrychus minutus* | 2 | EU |
|  |  |  |  |  |  |
|  | Ciconiidae | White Stork | *Ciconia ciconia* | 1 | EU |
|  |  |  |  |  |  |
| Anseriformes | Anatidae | Common Goldeneye | *Anas Clangula* | 1 | EU |
|  |  | Common Pochard | *Aythya ferina* | 4 | EU |
|  |  | Garganey | *Anas querquedula* | 1 | EU |
|  |  | Long-tailed Duck | *Anas hyemalis* | 2 | EU |
|  |  | Mandarin Duck | *Anas galericulata* | 3 | EU |
|  |  | Red-breasted Merganser | *Mergus serrator* | 5 | EU |
|  |  | Red-crested Pochard | *Netta rufina* | 1 | EU |
|  |  | Spot-billed Duck | *Anas poecilorhyncha* | 3 | AS |
|  |  |  |  |  |  |
|  |  | Canada Goose | *Branta canadensis* | 376 | EU |
|  |  | Egyptian Goose | *Alopochen aegyptiacus* | 100 | EU |
|  |  |  |  |  |  |
|  |  | Whooper Swan | *Cygnus cygnus* | 26 | EU |
|  |  |  |  |  |  |
| Falconiformes | Accipitridae | Black Kite | *Milvus migrans* | 1 | AS |
|  |  | Common Buzzard | *Buteo buteo* | 39 | EU |
|  |  | Eurasian Sparrowhawk | *Accipiter nisus* | 11 | EU |
|  |  | Long-legged Buzzard | *Buteo rufinus* | 1 | AS |
|  |  | Northern Goshawk | *Accipiter gentilis* | 18 | EU |
|  |  |  |  |  |  |
|  | Falconidae | Common Kestrel | *Falco tinnunculus* | 5 | EU |
|  |  | Lesser Kestrel | *Falco naumanni* | 1 | EU |
|  |  |  |  |  |  |
| Galliformes | Phasianidae | Common Pheasant | *Phasianus colchicus* | 2 | AS, EU |
|  |  | Common Quail | *Coturnix coturnix* | 15 | EU |
|  |  | Corn Crake | *Crex crex* | 1 | EU |
|  |  | Double-spurred Francolin | *Francolinus bicalcaratus* | 2 | AF |
|  |  | Grey Partridge | *Perdix perdix* | 3 | AS |
|  |  | Japanese Quail | *Coturnix japonica* | 1 | AS |
|  |  | Ptarmigan | *Lagopus muta* | 2 | EU |
|  |  | Ring-necked Pheasant | *Phasianus colchicus torquatus* | 1 | AS |
|  |  | Spotted Crake | *Porzana porzana* | 22 | EU |
|  |  | Stone Partridge | *Ptilopachus petrosus* | 1 | AF |
|  |  |  |  |  |  |
| Gruiformes | Rallidae | Common Coot | *Fulica atra* | 237 | EU |
|  |  | Moorhen | *Gallinula chloropus* | 220 | EU |
|  |  | Water Rail | *Rallus aquaticus* | 572 | EU |
|  |  |  |  |  |  |
|  |  |  |  |  |  |
| Charadriiformes | Haematopodiae | American Oystercatcher | *Haematopus palliatus* | 1 | SA |
|  |  | Magellanic Oystercatcher | *Haematopus leucopodus* | 20 | SA |
|  |  | Oystercatcher | *Haematopus ostralegus* | 88 | EU |
|  |  |  |  |  |  |
|  | Charadriidae | Common Ringed Plover | *Charadrius hiaticula* | 108 | EU |
|  |  | European Golden Plover | *Pluvialis apricaria* | 35 | EU |
|  |  | Great Ringed Plover | *Charadrius hiaticula* | 8 | EU |
|  |  | Grey Plover | *Pluvialis squatarola* | 19 | EU |
|  |  | Kentish Plover | *Charadrius alexandrinus* | 9 | AS |
|  |  | Little Ringed Plover | *Charadrius dubius* | 108 | EU |
|  |  | Northern Lapwing | *Vanellus vanellus* | 5 | EU |
|  |  | Southern Lapwing | *Vanellus chilensis* | 4 | SA |
|  |  |  |  |  |  |
|  | Scolopacidae | Baird's Sandpiper | *Calidris bairdii* | 24 | SA |
|  |  | Bar-tailed Godwit | *Limosa lapponica* | 68 | EU |
|  |  | Broad-billed Sandpiper | *Limicola falcinellus* | 4 | EU |
|  |  | Buff-breasted Sandpiper | *Tryngites subruficollis* | 1 | SA |
|  |  | Common Greenshank | *Tringa nebularia* | 34 | EU |
|  |  | Common Redshank | *Tringa totanus* | 26 | EU |
|  |  | Common Sandpiper | *Acitis hypoleucos* | 40 | EU |
|  |  | Common Snipe | *Gallinago gallinago* | 54 | AS, EU |
|  |  | Curlew Sandpiper | *Calidris ferruginea* | 621 | EU |
|  |  | Dotterel | *Charadrius morinellus* | 1 | EU |
|  |  | Dunlin | *Calidris alpina* | 882 | AS, EU, NA |
|  |  | Eurasian Curlew | *Numenius arquata* | 20 | EU |
|  |  | Far Eastern Curlew | *Numenius madagascariensis* | 11 | AS |
|  |  | Great Snipe | *Gallinago media* | 28 | EU |
|  |  | Green Sandpiper | *Tringa ochropus* | 93 | EU |
|  |  | Jack Snipe | *Gallinago media* | 26 | AF |
|  |  | Lesser Yellowlegs | *Tringa flavipes* | 6 | SA |
|  |  | Little Stint | *Tringa minuta* | 78 | EU |
|  |  | Northern Lapwing | *Vanellus vanellus* | 5 | AF, EU |
|  |  | Pectoral Sandpiper | *Calidris melanotos* | 2 | SA |
|  |  | Purple Sandpiper | *Calidris maritima* | 6 | EU |
|  |  | Red-necked Phalarope | *Phalaropus lobatus* | 2 | EU |
|  |  | Ruddy Turnstone | *Arenaria interpres* | 26 | EU,NA |
|  |  | Ruff | *Philomachus pugnax* | 91 | EU |
|  |  | Sanderling | *Calidris alba* | 11 | EU, SA |
|  |  | Spotted Redshank | *Tringa erythropus* | 9 | EU |
|  |  | Spotted Sandpiper | *Actitis macularia* | 1 | EU |
|  |  | Temminck's Stint | *Calidris temminckii* | 13 | EU |
|  |  | Terek Sandpiper | *Tringa cinerea* | 1 | AS |
|  |  | Two-banded Plover | *Charadrius falklandicus* | 54 | SA |
|  |  | Whimbrel | *Numeniusphaeopus* | 7 | EU |
|  |  | White-rumped Sandpiper | *Calidris fuscicolli* | 48 | SA |
|  |  | Wood Sandpiper | *Tringa glareola* | 220 | AS, EU |
|  |  | Woodcock | *Scolopax rusticola* | 6 | EU |
|  |  |  |  |  |  |
|  | Thinocoridae | Least Seedsnipe | *Thinocorus rumicivorus* | 2 | SA |
|  |  |  |  |  |  |
|  | Chionididae | Snowy Sheathbill | *Chionis alba* | 1 | ANT |
|  |  |  |  |  |  |
|  | Stercorariidae | Brown Skua | *Stercorarius lonnbergi* | 4 | ANT |
|  |  | Great Skua | *Stercorarius skua* | 2 | EU |
|  |  | Parasitic Jaeger | *Stercorarius parasiticus* | 5 | EU |
|  |  | Pomarine Skua | *Stercorarius pomarinus* | 1 | EU |
|  |  | South Polar Skua | *Stercorarius maccormicki* | 2 | ANT |
|  |  |  |  |  |  |
|  | Laridae | Arctic Tern | *Sterna paradisaea* | 4 | EU |
|  |  | Black-tailed Gull | *Larus crassirostris* | 9 | AS |
|  |  | Common Tern | *Sterna hirundo* | 18 | EU |
|  |  | Kittiwake | *Rissa tridactyla* | 58 | EU |
|  |  | Lesser Black-backed Gull | *Larus fuscus* | 1402 | EU |
|  |  | Mew Gull | *Larus canus canus* | 1 | EU |
|  |  | Yellow-legged Gull | *Larus cachinnans* | 4 | EU |
|  |  |  |  |  |  |
|  | Alcidae | Atlantic Puffin | *Fratercula arctica* | 38 | EU |
|  |  | Black Guillemot | *Cepphus grylle* | 3 | EU |
|  |  | Razorbill | *Alca torda* | 49 | EU |
|  |  |  |  |  |  |
| Columbiformes | Columbidae | Adamawa Turtle-Dove | *Streptopelia hypopyrrha* | 64 | AF |
|  |  | Black-billed Wood-Dove | *Turtur abyssinicus* | 16 | AF |
|  |  | Laughing Dove | *Streptopelia senegalensis* | 16 | AF |
|  |  | Red-billed Wood Dove | *Turtur afer* | 3 | AF |
|  |  | Rufous Turtle-Dove | *Streptopelia orientalis* | 2 | AS |
|  |  | Stock Dove | *Columba oenas* | 2 | AS, EU |
|  |  | Wood Pigeon | *Columba palumbus* | 6 | EU |
|  |  |  |  |  |  |
| Culculiformes | Cuculidae | Senegal Coucal | *Centropus senegalensis* | 2 | AF |
|  |  |  |  |  |  |
| Strigiformes | Tytonidae | Barn Owl | *Tyto alba* | 3 | EU |
|  |  |  |  |  |  |
|  | Strigidae | Tawny Owl | *Strix aluco* | 2 | EU |
|  |  | Eurasian Scops-Owl | *Otus scops* | 1 | AS |
|  |  |  |  |  |  |
| Caprimulgiformes | Caprimulgidae | Eurasian Nightjar | *Caprimulgus europaeus* | 3 | AS |
|  |  |  |  |  |  |
| Coliiformes | Coliidae | Speckled Mousebird | *Colius striatus* | 24 | AF |
|  |  |  |  |  |  |
| Coraciiformes | Alcedinidae | Common Kingfisher | *Alcedo atthis* | 16 | AF, EU |
|  |  | Grey-headed Kingfisher | *Alcedo leucocephala* | 3 | AF |
|  |  |  |  |  |  |
|  | Meropidae | Red-throated Bee-eater | *Merops bulocki* | 18 | AF |
|  |  |  |  |  |  |
|  | Bucerotes | Red-billed Wood-hoopoe | *Promerops purpureus* | 2 | AF |
|  |  |  |  |  |  |
| Piciformes | Indicatoridae | Greater Honeyguide | *Indicator indicator* | 16 | AF |
|  |  | Lesser Honeyguide | *Indicator minor* | 1 | AF |
|  |  | Willcocks's Honeyguide | *Indicator willcocksi* | 1 | AF |
|  |  |  |  |  |  |
|  | Ramphastidae | Bearded Barbet | *Lybius dubius* | 3 | AF |
|  |  | Speckled Tinkerbird | *Pogoniulus scolopaceus* | 2 | AF |
|  |  | Veillot's Barbet | *Lybius veilloti* | 5 | AF |
|  |  | White-headed Barbet | *Lybius leucocephalus* | 2 | AF |
|  |  | Yellow-fronted Tinkerbird | *Pogoniulus chrysoconus* | 21 | AF |
|  |  |  |  |  |  |
|  | Picidae | Fine-spotted Woodpecker | *Campethera punctuligera* | 1 | AF |
|  |  | Great Spotted Woodpecker | *Dendrocopus major* | 9 | EU |
|  |  | Grey Woodpecker | *Dendropicos goertae* | 3 | AF |
|  |  | Japanese Pygmy Woodpecker | *Dendrocopos kizuki kizuki* | 3 | AS |
|  |  | Wryneck | *Jynx torquilla* | 9 | AF, EU |
|  |  |  |  |  |  |
| Passeriformes | Sylviidae | African Moustached Warbler | *Melocichla mentalis* | 1 | AF, EU |
|  |  | Aquatic Warbler | *Acrocephalus paludicola* | 5 | EU |
|  |  | Arctic Warbler | *Phylloscopus borealis* | 3 | AS |
|  |  | Blackcap | *Sylvia aricapilla* | 1 | EU |
|  |  | Blyth's Reed Warbler | *Acrocephalus dumetorum* | 1 | AS |
|  |  | Bonelli’s Warbler | *Phylloscopus bonelli* | 1 | EU |
|  |  | Booted Warbler | *Hippolais caligata* | 1 | EU |
|  |  | Cetti’s Warbler | *Cettia cetti* | 6 | AS, EU |
|  |  | Chiffchaff | *Phylloscopus collybita* | 179 | EU |
|  |  | Common Whitethroat | *Sylvia communis* | 114 | AF, EU |
|  |  | Dusky Warbler | *Phylloscopus fuscatus* | 1 | EU |
|  |  | Garden Warbler | *Sylvia borin* | 164 | AF, EU |
|  |  | Grasshopper Warbler | *Locustella naevia* | 97 | AS, EU |
|  |  | Green Crombec | *Sylvietta virens* | 1 | AF, EU |
|  |  | Icterine Warbler | *Hippolais icterina* | 4 | EU |
|  |  | Lesser Whitethroat | *Sylia curruca* | 93 | AS, EU |
|  |  | Marsh Warbler | *Acrocephalus palustris* | 32 | EU |
|  |  | Moustached Grass-Warbler | *Melocichla mentalis* | 1 | AF |
|  |  | Northern Crombec | *Sylvietta brachyura* | 1 | AF |
|  |  | Reed Warbler | *Acrocephalus scirpaceus* | 185 | EU |
|  |  | Savi’s Warbler | *Locustella luscinioides* | 7 | EU |
|  |  | Sedge Warbler | *Acrocephalus schoenobaenus* | 109 | EU |
|  |  | Willow Warbler | *Phylloscopus trochilus* | 127 | AF, EU |
|  |  | Wood Warbler | *Phylloscopus sibilatrix* | 1 | EU |
|  |  | Yellow-browed Warbler | *Phylloscopus inornatus* | 3 | AS |
|  |  |  |  |  |  |
|  | Passeridae | Tree Sparrow | *Passer motanus* | 5 | AS, EU |
|  |  | Bush Petronia | *Petronia dentata* | 2 | AF |
|  |  | Chestnut-crowned Sparrow-Weaver | *Plocepasser superciliosus* | 5 | AF |
|  |  | Grey-headed Sparrow | *Passer griseus* | 2 | AF |
|  |  | House Sparrow | *Passer domesticus* | 5 | AS |
|  |  |  |  |  |  |
|  | Monarchidae | African Paradise Flycatcher | *Terpsiphone viridis* | 4 | AF |
|  |  |  |  |  |  |
|  | Turdidae | African Thrush | *Turdus pelios* | 58 | AF, EU |
|  |  | Eurasian Blackbird | *Turdus merula* | 173 | EU |
|  |  | Bluethroat | *Luscinia svecica* | 39 | AS, EU |
|  |  | Fieldfare | *Turdus pilaris* | 162 | EU |
|  |  | Mistle Thrush | *Turdus viscivorus* | 2 | EU |
|  |  | Redwing | *Turdus iliacus* | 150 | EU |
|  |  | Ring Ouzel | *Turdus torquatus* | 1 | EU |
|  |  | Robin | *Erithacus rubecula* | 181 | EU |
|  |  | Song Thrush | *Turdus philomelus* | 166 | EU |
|  |  | Whinchat | *Saxicola rubetra* | 7 | AF, EU |
|  |  |  |  |  |  |
|  | Incertae Sedis | African Blue Flycatcher | *Elminia longicauda* | 2 | AF |
|  |  |  |  |  |  |
|  | Timaliidae | Blackcap Babbler | *Turdoides reinwardtii* | 187 | AF |
|  |  | Brown Babbler | *Turdoides plebejus* | 1 | AF |
|  |  |  |  |  |  |
|  | Malaconotidae | Black-crowned Tchagra | *Tchagra senegalus* | 8 | AF |
|  |  | Common Gonolek | *Laniarius barbarus* | 10 | AF |
|  |  | Sulphur-breasted Bushshrike | *Telophorus sulfureopectus* | 6 | AF |
|  |  |  |  |  |  |
|  | Cisticolidae | Bleating Warbler | *Camaroptera brachyura* | 4 | AF |
|  |  | Grey-backed Camaroptera | *Camaroptera brevicaudata* | 11 | AF |
|  |  | Oriole Warbler | *Hypergerus atriceps* | 5 | AF |
|  |  | Rock-loving Cisticola | *Cisticola emini* | 2 | AF |
|  |  | Winding Cisticola | *Cisticola galactotes* | 10 | AF |
|  |  | Yellow-breasted Apalis | *Apalis flavida* | 2 | AF |
|  |  |  |  |  |  |
|  | Pipridae | Bronze Manakin | *Lonchura cucullata* | 2 | AF |
|  |  |  |  |  |  |
|  | Regulidae | Firecrest | *Regulus ignicapilla* | 76 | EU |
|  |  | Goldcrest | *Regulus regulus* | 119 | EU |
|  |  |  |  |  |  |
|  | Malaconotidae | Northern Puffback Shrike | *Dryoscopus gambensis* | 1 | AF |
|  |  |  |  |  |  |
|  | Viduidae | Pin-tailed Whydah | *Vidua macroura* | 1 | AF |
|  |  | Village Indigobird | *Vidua chalybeata* | 4 | AF |
|  |  |  |  |  |  |
|  | Platysteiridae | Brown-throated Wattle-eye | *Platysteira cyanea* | 8 | AF |
|  |  | Senegal Batis | *Batis senegalensis* | 5 | AF |
|  |  |  |  |  |  |
|  | Alaudidae | Sky Lark | *Alauda gulgula* | 171 | AS, EU |
|  |  | Wood Lark | *Lullula arborea* | 5 | EU |
|  |  | Sun Lark | *Galerida modesta* | 1 | AF |
|  |  |  |  |  |  |
|  | Hirundinidae | Barn Swallow | *Hirundo rustica* | 5 | AS |
|  |  |  |  |  |  |
|  | Motacillidae | Grey Wagtail | *Motacilla cinerea* | 32 | EU |
|  |  | Meadow Pipit | *Anthus pratensis* | 137 | EU |
|  |  | Olive-backed Pipit | *Anthus hodgsoni* | 3 | AS |
|  |  | Pied Wagtail | *Motacilla alba* | 8 | EU |
|  |  | Red-throated Pipit | *Anthus cervinus* | 2 | AS |
|  |  | Tree Pipit | *Anthus trivialis* | 17 | AF, AS, EU |
|  |  | Water Pipit | *Anthus spinoletta* | 1 | EU |
|  |  | Yellow-throated Longclaw | *Macronyx croceus* | 3 | AF, AS, EU |
|  |  |  |  |  |  |
|  | Campephagidae | Red-shouldered Cuckoo-shrike | *Campephaga phoenicea* | 1 | AF |
|  |  |  |  |  |  |
|  | Pycnonotidae | Brown-eared Bulbul | *Ixos amaurotis* | 17 | AS |
|  |  | Common Bulbul | *Pycnonotus barbatus* | 72 | AF |
|  |  | Little Greenbul | *Andropadus virens* | 1 | AF |
|  |  | Simple Leaflove | *Chlorocichla simplex* | 1 | AF |
|  |  | Yellow-throated Leaflove | *Chlorocichla flavicollis* | 7 | AF |
|  |  |  |  |  |  |
|  | Laniidae | Great Grey Shrike | *Lanius excubitor* | 4 | EU |
|  |  |  |  |  |  |
|  | Troglodytidae | Winter Wren | *Troglodytes troglodytes* | 88 | EU |
|  |  |  |  |  |  |
|  | Prunellidae | Hedge Accentor | *Prunella modularis* | 123 | EU |
|  |  |  |  |  |  |
|  | Muscicapidae | Common Nightingale | *Luscinia megarhynchos* | 74 | AF, EU |
|  |  | Common Redstart | *Phoenicurus phoenicurus* | 28 | EU |
|  |  | Common Stonechat | *Saxicola rubicola* | 5 | AS, EU |
|  |  | Daurian Redstart | *Phoenicurus auroreus* | 2 | AS |
|  |  | Familiar Chat | *Cercomela familiaris* | 21 | AF |
|  |  | Grey-winged Robin-Chat | *Cossypha polioptera* | 1 | AF |
|  |  | Mocking Cliff-Chat | *Thamnolaea cinnamomeiventris* | 7 | AF |
|  |  | Northern Anteater-Chat | *Myrmecocichla aethiops* | 2 | AF |
|  |  | Northern Black-Flycatcher | *Melaenornis edolioides* | 4 | AF |
|  |  | Northern Wheatear | *Oenanthe oenanthe* | 5 | EU |
|  |  | Pale Flycatcher | *Bradornis pallidus* | 2 | AF |
|  |  | Pied Flycatcher | *Fidecula hypoleuca* | 19 | EU |
|  |  | Spotted Flycatcher | *Muscicapa striata* | 2 | EU |
|  |  | Snowy-crowned Robin-Chat | *Cossypha niveicapilla* | 20 | AF |
|  |  | Whinchat | *Saxicola rubetra* | 7 | AF, EU |
|  |  | White-crowned Robin-Chat | *Cossypha albicapilla albicapilla* | 4 | AF |
|  |  | White-fronted Black-Chat | *Myrmecocichla albifrons* | 1 | AF |
|  |  |  |  |  |  |
|  | Paridae | Bearded Tit | *Panurus biarmicus* | 40 | EU |
|  |  | Blue Tit | *Parus caerleus* | 188 | EU |
|  |  | Coal Tit | *Parus ater* | 4 | EU |
|  |  | Great Tit | *Parus major* | 85 | AS, EU |
|  |  | Long-tailed Tit | *Aegithalos caudatus* | 68 | AS, EU |
|  |  | Marsh Tit | *Poecile palustris* | 4 | AS, EU |
|  |  | Turkestan Tit | *Parus bokharensis* | 5 | AS |
|  |  | Willow Tit | *Parus montanus* | 1 | EU |
|  |  | Yellow-breasted Tit | *Cyanistes flavipectus* | 5 | AS |
|  |  |  |  |  |  |
|  | Certhiidae | Short-toed Treecreeper | *Certhia brachydactyla* | 5 | EU |
|  |  |  |  |  |  |
|  | Nectariniidae | Green-headed Sunbird | *Nectarinia verticalis* | 4 | AF |
|  |  | Olive Sunbird | *Nectarinia olivacea* | 4 | AF |
|  |  | Olive-backed Sunbird | *Nectarinia jugularis* | 4 | AF |
|  |  | Scarlet-chested Sunbird | *Nectarinia senegalensis* | 7 | AF |
|  |  | Variable Sunbird | *Nectarinia venusta* | 2 | AF |
|  |  |  |  |  |  |
|  | Zosteropidae | African Yellow White-eye | *Zosterops senegalensis* | 14 | AF |
|  |  |  |  |  |  |
|  | Emberizidae | Black-faced Bunting | *Emberiza spodocephala* | 2 | AS |
|  |  | Chestnut Bunting | *Emberiza rutila* | 3 | AS |
|  |  | Cinnamon-breasted Rock Bunting | *Emberiza tahapisi* | 4 |  |
|  |  | Corn Bunting | *Emberiza calandra* | 1 | EU |
|  |  | Ortolan Bunting | *Emberiza hortulana* | 1 | EU |
|  |  | Reed Bunting | *Emberiza schoeniclus* | 99 | EU |
|  |  | Rock Bunting | *Emberiza cia* | 4 | AF, AS |
|  |  | Rustic Bunting | *Emberiza rustica* | 2 | AS |
|  |  | Snow Bunting | *Plectrophenax nivalis* | 1 | EU |
|  |  | Tristram's Bunting | *Emberiza tristrami* | 1 | AS |
|  |  | Yellow-throated Bunting | *Emberiza elegans* | 3 | AS |
|  |  |  |  |  |  |
|  | Fringillidae | Common Rosefinch | *Carpodacus erythrinus* | 1 | EU |
|  |  | Eurasian Bullfinch | *Pyrrhula pyrrhula* | 25 | EU |
|  |  | European Goldfinch | *Carduelis carduelis* | 1 | EU |
|  |  |  |  |  |  |
|  | Estrildidae | Black-bellied Firefinch | *Lagonosticta rara* | 1 | AF |
|  |  | Black-rumped Waxbill | *Estrilda troglodytes* | 4 | AF |
|  |  | Brambling | *Fringilla montifringilla* | 52 | EU |
|  |  | Bullfinch | *Pyrrhula pyrrhula* | 25 | EU |
|  |  | Chaffinch | *Fringilla colebs* | 69 | AS, EU |
|  |  | Goldfinch | *Carduelis carduelis* | 1 | EU |
|  |  | Greenfinch | *Carduelis chloris* | 5 | EU |
|  |  | Grey-headed Oliveback | *Nesocharis capistrata* | 1 | AF |
|  |  | Lavender Waxbill | *Estrilda caerulescens* | 16 | AF |
|  |  | Linnet | *Carduelis cannabina* | 1 | EU |
|  |  | Red-cheeked Cordon-bleu | *Uraeginthus bengalus* | 13 | AF |
|  |  | Rock Firefinch | *Lagonosticta sanguinodorsalis* | 20 | AF |
|  |  | Senegal Firefinch | *Lagonosticta senegala* | 3 | AF |
|  |  |  |  |  |  |
|  | Ploceidae | Black-necked Weaver | *Ploceus nigricollis* | 27 | AF |
|  |  | Black-winged Bishop | *Euplectes hordeaceus* | 26 | AF |
|  |  | Heuglin's Masked-Weaver | *Ploceus heuglini* | 10 | AF |
|  |  | Little Weaver | *Ploceus luteolus* | 3 | AF |
|  |  | Northern Red Bishop | *Euplectes orix* | 35 | AF |
|  |  | Speckle-fronted Weaver | *Sporopipes frontalis* | 8 | AF |
|  |  | Village Weaver | *Ploceus cucullatus* | 50 | AF |
|  |  | Vitelline Masked-Weaver | *Ploceus vitellinus* | 18 | AF |
|  |  | Yellow-crowned Bishop | *Euplectes afer* | 1 | AF |
|  |  |  |  |  |  |
|  | Sturnidae | Common Starling | *Sturnus vulgaris* | 203 | AS, EU |
|  |  | Grey Starling | *Sturnus cineraceus* | 15 | AS, EU |
|  |  | Purple Glossy-Starling | *Lamprotornis purpureus* | 1 | AF |
|  |  | Violet-backed Starling | *Cinnyricinclus leucogaster* | 1 | AF |
|  |  |  |  |  |  |
|  |  |  |  |  |  |
|  | Corvidae | Black-billed Magpie | *Pica hudsonia* | 2 | AS |
|  |  | Carrion Crow | *Corvus corone* | 14 | EU |
|  |  | Eurasian Magpie | *Pica pica* | 4 | AS, EU |
|  |  | Eurasian Jackdaw | *Corvus monedula* | 13 | AS, EU |
|  |  | Eurasian Jay | *Garrulus glandarius* | 1 | EU |
|  |  | Jungle Crow | *Corvus levaillantii* | 22 | AS |
|  |  | Rook | *Corvus frugilegus* | 1 | EU |
|  |  |  |  |  |  |
|  |  |  |  |  |  |
|  |  |  |  |  |  |
|  |  |  |  |  |  |
|  |  |  |  |  |  |
|  |  |  |  |  |  |
|  |  |  |  |  |  |
